# Losing persons: the pastoral imperative for affirming continued personhood for those living with dementia

**DOI:** 10.1080/11287462.2025.2532920

**Published:** 2025-07-21

**Authors:** Stephen R. Milford

**Affiliations:** a Institute for Biomedical Ethics, Basel University, Basel, Switzerland; b Department of Theology, North-West University, Potchefstroom, South Africa

**Keywords:** Dementia, human personhood, Dworkin and Dresser, advanced care directives, pastoral care

## Abstract

Dementia is responsible for untold suffering, most significant is the fear that someone will lose themselves. This fear raises very serious pastoral questions: Who is the person living with dementia? Are they the same person they have always been or someone new? In either case, how do we treat them? Using the well-known case of Margo and the discussions between Dworkin and Dresser around advanced care directives, this article radically challenges the standard psychology view of personhood as being pastorally unhelpful in dementia cases. We argue that a relational view of personhood is not only epistemologically consistent but better suited to the pastoral challenge presented by dementia than that of the standard psychological view. While dementia represents the loss of cognitive abilities, and in many cases an entire change in personality, it does not represent either the loss of a person nor a change of personhood. Through dementia a person remains a person because they are personally related to by the same community of persons who have always loved them. This normative framework offers those living with dementia, their community, and their carers with a coherent, yet pastorally helpful response to the existential questions raised by dementia.

## Introduction

Dementia affects more than 55 million people globally, with 10 million new cases each year (WHO, 2023). Apart from the staggering loss of life (currently the 7th leading cause of death), the immense suffering caused, or even the economic disaster – costing the global economy 1.3 trillion dollars annually, is the assault on the very identity/personhood of those living with dementia. This, perhaps more than anything else, is the “terror” (Behuniak, 2011) of a dementia diagnosis: that one will lose themselves, that one will cease to be the persons they once were (Macklin, 1983). The “someone else problem” – as DeGrazia (1999) phrases it – creates numerous subsequent challenges. Questions arise as to how those living with dementia are to be treated, how their prior wishes are to be respected, and the role of their family in deciding their last moments. It is these questions, particularly as they relate to the moral legitimacy of advanced care directives (ACDs), that have plagued bioethics for decades (Dale, 2021; DeGrazia, 1999; Demarco & Lipuma, 2016; Gligorov & Vitrano, 2011).

Yet what has often gone unremarked is the pastoral crisis that is created and the consequential pastoral care required. While the term *pastoral care* carries with it religious connotations in line with its historical roots (Clebsch & Jaekle, 1983), in recent decades it has been appropriated into secular discourse (Schuhmann & Damen, 2018). Used in this article, the term “pastoral” refers to the broader concern of human flourishing: those aspects of human existence that go beyond simply being alive to experiencing the fullness of humanity. This includes mental and physical states, happiness, life satisfaction, meaning and purpose, character and virtue, etc. (VanderWeele, 2017). Understood this way, pastoral care refers to the care given to those living with dementia beyond the physical treatment provided. It is the emotional, spiritual, and ethical guidance provided to those living with dementia and plays a crucial role in supporting patients and their families through the entire process of living and dying with dementia. While dementia certainly brings with it health crises, it is often this broader pastoral crisis brought on by the apparent assault on one’s identity that is brought into sharp relief. Those living with dementia might ask themselves: Will I lose myself? Will I become someone else entirely, or worse, no one at all?

This fear extends beyond the person who develops dementia to their community. The phrase: “They are not who they once were” (Day, 2017) is the commonplace lament of family and friends. For this community, a dementia diagnosis entails a life lived “constantly in a state of grieving” (Addo, 2020; see also Davidson, 2015). This itself presents a related pastoral challenge. What do we say to family and friends? Do we tell them that they will lose their loved one to a “living death” (Hill, 2008; see also Lushin, 1990), a kind of “death before death” (Cohen & Eisdorfer, 2001) as the media and others have sometimes portrayed those living with dementia. This “zombification” (Schweda & Jongsma, 2022) of dementia has been rightly criticised (Behuniak, 2011; Peel, 2014; Schweda & Jongsma, 2022). While much work has been done to develop constructive caring practices for those living with dementia, little guidance has been provided for a suitable normative framework that is pastorally helpful.

This article will consider the implications of different frameworks of personhood for the pastoral care of both those living with dementia and their community. We will begin with a brief discussion on Margo, a well-known case of dementia in which the question of personhood becomes central. We will then move to critique the “Standard View” (Olson, 1997) of personhood and to consider what is here offered as a better approach: the relational.

Before we begin, a few words of caution are merited. First, ACD, ACP (Advanced Care Planning) and AD (Advance Directives) are widely debated within the context of dementia with distinct operationalisations and definitions across countries (Rietjens et al., 2017; van der Steen et al., 2024). As our focus in this project is not about the ethics of ACD, ACP or ADs we will refrain from in-depth discussions around this topic here. We use the case of Margo and ACDs merely as a springboard for deeper discussions on pastorally helpful conceptions of personhood for those living with dementia.

Second, we make the assumption that inclusion into the category of person is valuable in itself as it bestows certain rights (Kelsey, 2009). We will avoid tangents that seek to debate the basis of these rights (dignity, consciousness, legality, human DNA etc.) and simply take for granted that it is persons everywhere (human or not) that have value and basic rights.

Third, the exact definition of human personhood has been debated for millennia and there is little evidence of an imminent consensus. Our project here will not provide this illusive consensus. Rather, we are seeking to compare different views of personhood for their pastoral implications. It is very possible that personhood is the kind of concept that has different definitions for different contexts. For example, a legal person may not be the same as a metaphysical person. During our project here we will argue for the pastoral superiority of a relational view of personhood.

Fourth, for many readers this philosophical approach is new. The literature on relational personhood is extensive, and an exhaustive discussion is beyond our scope here. Therefore, we will refrain from an in-depth critique on this well-established philosophical approach to personhood. The interested reader is encouraged to consider the vast literature on this point (McFadyen, 1990; Milford, 2020b; Milford & Shaw, 2023; Schaab, 2013; Shults, 2003). We acknowledge that the relational construction, like all constructions, is not without its limitations, some of which we have addressed elsewhere (Milford, 2020b). Our discussions on relational personhood should not be taken as an exhaustive critical discussion, but rather a very rudimentary overview to help the reader situate relational personhood within the context of philosophical discussions on personhood and how this established construct is particularly relevant to the pastoral questions raised by dementia.

## Who is Margo Really?

Andrew D. Firlik (1991) describes a case he encountered early in his training which involved a 55 year-old woman called Margo. Margo developed dementia. While she was in advanced stages of dementia, she appeared to be quite content which led Firlik to ask who Margo actually was:

Despite her illness, or maybe somehow because of it, Margo is undeniably one of the happiest people I have known. There is something graceful about the de-generation her mind is undergoing, leaving her carefree, always cheerful. Do her problems, what – ever she may perceive them to be, simply fail to make it to the worry centers of her brain? How does Margo maintain her sense of self? When a person can no longer accumulate new memories as the old rapidly fade, what remains? Who is Margo? (1991, p. 201)

Margo has since become the centre of a number of debates around the question of dementia and human personhood (Dale, 2021; Nys, 2013; Rich, 1997). In particular is a dialogue between Dworkin (Dworkin, 1995) and Dresser (1995) about the legitimacy of ACDs in the context of dementia and human identity. These discussions are relevant here as they directly address the “someone else problem.” There is, therefore, value in spending a bit of time considering this debate.

The central concern may be summarised as such: Dworkin supposes that Margo executed an ACD prior to her dementia diagnosis. In this directive, Margo stated that she should not receive treatment for any serious, life-threating disease she might contract. Furthermore, in such an event “she should be killed as soon and as painlessly as possible” (Dworkin, 1995, p. 226). The question is: Should we honour this request? As Dale puts it: “arguably in her current state Margo is happy and therefore should be given non-invasive treatments, like oral antibiotics, to extend her life if she contracts pneumonia” (Dale, 2021).

Margo’s cases brings to the fore two different views of those living with dementia (Parens, 2013). On the one hand, one could consider Margo pre-dementia (Margo PD) to be the same person as Margo with dementia (Margo WD) – the diseased view. Or one could argue that Margo PD is not the same person as Margo WD: she is a distinct person – the different view. Dworkin adopts the former view. According to Dworkin (1995) humans have two important interests (see also Dale, 2021; Parens, 2013; Rich, 1997). First, there are experiential interests: the desire to experience pleasure and avoid pain. For each individual, these experiences may be different. One may enjoy opera or certain sports. It is not that one experience is right and the other wrong, or that one is mistaken for enjoying the one and not the other. Rather these experiences are pleasurable “because and when they feel good” (Dworkin, 1995, p. 202). The second interest is that of critical interests. These interests give life meaning across time. They determine, for each individual, what it means to live a good life. They are the interests that guide an individual’s life and are what “strike them as not only good at the moment but in character for them” (Dworkin, 1995, p. 202). An example of these interests may be the interest to have close relationships with friends and family, to live a life of dignity (whatever that may mean for an individual) and to live a life according to their own wishes (autonomy).

To Dworkin, critical interests are more important than experiential interests. A life lived with meaning across time, should outweigh the desire to experience pleasure and avoid pain in the moment. This provides a coherent narrative across one’s life (Rich, 1997; consider also Ricoeur, 1990). Rhoden asserts: “Considering the patient only in the immediate present divides the patient from her past, her history, her values, and her relationships – from all those things that make her human” (in Rich, 1997, n. 18). Consequently, while Margo WD may be happy, her present experiential interests do not outweigh her extended critical interests. Her ACD should be honoured (Dale, 2021; Dworkin, 1995; Parens, 2013; Rich, 1997). Ignoring Margo’s ACD would be “an unacceptable form of moral paternalism” (Dworkin, 1995, p. 231) because it enforces a conclusion to Margo’s life that she explicitly wished to avoid. It ignores the “strongest living articulation of her autonomy” (Dale, 2021, p. 3).

Dresser disagrees. She questions whether Margo PD is the same person as Margo WD:

substantial memory loss and other psychological changes may produce a new person, whose connection to the earlier one could be less strong, indeed, could be no stronger than that between you and me. Subscribers to this view of personal identity can argue that Margo's earlier choices lack moral authority to control what happens to Margo the dementia patient. (Dresser, 1995, p. 35)

Consequently, Margo’s prior ACD should be ignored, and she should be given lifesaving anti-biotics to treat pneumonia.

There are serious challenges to both positions. Dworkin makes it impossible for a sufferer of dementia to have their ACD amended on the basis that they must always honour the critical interests they formally chose to dictate the narrative of their life. Dresser, on the other hand, argues that an ACD is of little significance, as dementia is the sort of condition that creates a new person. The wishes of the old person cannot be forced upon the wishes of the new person any more than one’s own wishes can be forced on another.

While these discussions have played out in the context of ACD legitimacy and have serious repercussions for both life-extending treatment as well as life-ending practices (euthanasia), they are particularly important for the manner in which we provide pastoral care to both those living with dementia and their community. The solutions offered by authors such as Dworkin and Dresser are based on a particular normative understanding of human personhood which feeds into their understanding of the implications of dementia on human personhood and consequently their suggested practices. Drawing on their arguments for the legality (or illegality) of ACDs can assist us in deciding how we pastorally respond to people living with dementia.

Dworkin can be interpreted as affirming an individualistic narrative view of personhood. That is to say: one’s personhood is not established minute my minute, but across their individual life-history (Rich, 1997; Ricoeur, 1990). One’s values and character at certain times of their life – when they are most cognitively coherent – have implications for other times of their life – when their cognitive abilities diminish. At the end of their life, a person with dementia may “have essentially lost touch with reality” (Rich, 1997, p. 143). To live in this “virtual reality” (Rich, 1997, pp. 142–144), so Rich argues, would “repulse” (Rich, 1997, p. 143) most people, no matter how pleasant the experience might be. Pleasure and pain should be denigrated to a second-order interest. They should be ignored in the face of critical interests: a life lived for deeper meaning. Using this model of personhood one finds oneself in the absurd position of arguing that once the old personhood dies, the “predementia individual retains posthumous rights over her [living] body that she acquired through historical embodiment in that body” (Persad, 2019, p. 249; see also DeGrazia, 1999).

To Dresser, human personhood is the consequence of the particular psychological or cognitive capacities a human being inhabits at a particular point in time. As these capacities change, so too does the person. Consequently, one may start out as one person and die an entirely different person as one’s personality changes over time. When does this change take place? At what point did Margo become a different person? Was there a clean break: Did Margo go to bed the old Margo and wake up the next morning a new person who happens to bear the same name? Was it a transition: At some stage Margo was a bit of the new Margo and a bit of the old? In which case there must have been a time when Margo was half-and-half (two half persons merged together?) until she become a new Margo. If this is the case, can one really argue that personhood lends itself to degrees (Perring, 1997)? If so, what would the implications be to persons elsewhere if we were to say some are only partly persons, and others full persons?

While these normative frameworks of personhood are important for ACDs, they also have direct bearings on pastoral care. Setting aside the logical legitimacy of these frameworks (which we will shortly critique), each framework encourages a different response to both those living with dementia and their community. When we consider that it is common to associate personhood with something valuable, the pastoral consequences of frameworks for personhood become significant. To cease to be a person is to cease to have distinct value, dignity, and rights. Should we choose a Dworkin’s framework, we would have to explain to the person living with dementia and their family that even if there is joy, past critical interests must now dictate what happens to the person living with dementia. If we chose Dresser’s approach, we would have to say to someone living with dementia that once they have contracted dementia they are in the process of becoming someone entirely different. Their family will be in a constant state of grief (Davidson, 2015), like a “funeral that never ends’ (Smith, 1992, p. 49), as they are replaced bit by bit by someone completely different. To the family, we would need to explain that in the end they will have to carry the burden of caring for an entirely strange person with no connection to the former person save for the same proper noun and a few rudimentary biological features.

While these responses are sometimes employed, and certainly may at times feel true, there is reason to question both the philosophical coherence and the pastoral validity of either framework.

## From the standard view to the relational

What exactly is a person? This question has been debated *ad infinitum* for thousands of years. However, there has been a recurrent theme that has come to be known as the “Standard View” (Olson, 1997). Starting as far back as Aristotle who defined the human being as the “rational animal” (1905, p. 1 2, 1523a7-18), the notion that personhood is connected with some sort of persistent psychological or cognitive continuity is widespread (Lockwood, 1988; Manninen, 2014; McMahan, 2014; Stone, 1987). Locke, for example, argues that “*person* stands for … a thinking intelligent being, that has reason and reflection, and can consider itself as itself, the same thinking thing, in different times and places; which it does only by that consciousness which is inseparable from thinking, and, as it seems to be, essential to it” (Locke, 2004, p. 2. XXXI.9). The main psychological features associated with personhood have traditionally been rationality, morality, and a self-reflective consciousness. Those beings that possess these attributes may be considered persons. The failure to process these characteristics results in removal from the category of person, with all the entailed consequences.

Both Dworkin and Dresser affirm this view of personhood. To Dworkin, the rational Margo PD is a person because she is rational. She has the capacity to think clearly and to decide for herself. Therefore, she has the rights of persons, particularly the right to autonomy (in this case precedent/prospective autonomy) and can exercise this right over her body when its competency is in question. Dresser, like Dworkin, agrees that psychological characteristics are the essence of personhood, but unlike Dworkin, she argue that as these change, so too does the person. Margo PD is a particular person (a rational person perhaps?), while Margo WD is a distinctly different person (a happy person?). Nevertheless, both affirm that it is the possession of certain psychological or cognitive traits that grounds personhood.

While being widespread, the Standard View is not without its limitations. We have taken these up elsewhere in depth (*authors work redacted for anonymity*). In brief, the Standard View removes huge swaths of the human population from the category of persons: early infants, those with mental challenges, and of course many who live with dementia. These humans lack certain rationality, self-reflection, or moral capacity and consequently are not considered persons. This is the central tenet of animalism (founded on the Standard View) and the ultimate conclusion of DeGrazia (1999) who contends that during advanced stages of dementia the person disappears, and yet their identity remains in the same way as any non-person animal’s identity remains (McMahan, 2002; Olson, 1997).

This is highly problematic. As we noted in our introduction, it is persons (not human animals) that have distinct universal, inalienable and unqualified value, dignity, along with the associated rights. To remove someone living with dementia from the category of persons, therefore, would have profound consequences. Apart from legal and ethical consequences – such as the legitimacy of ACDs – the pastoral consequences would be dire. Who would dare tell the wife of someone living with dementia that her spouse is not a person anymore when they are confused and scared?

Elsewhere we have argued that that those who affirm the Standard View have conflated personality with personhood (Milford & Shaw, 2023). To have a personality does not entail that one is a person. Non-human entities (animate and inanimate) are said to have a personality. A beloved piece of furniture, for example, may be said to have a distinct personality, and the same may be said of an animal such as a dog. While these are of course metaphorical uses of the term personality they point to the underlying common notion that personality is associated with individuality or uniqueness. Here a family pet is valued (it is loved) for its uniqueness. However, while it may be true that humans are unique individuals, this is not the bases of their value. Humans are not valued because they are individually unique, but because each is unsubstitutable for another. The basis of this unsubstitutability is not their individualism, but the unique manner in which they are personally loved and valued by other persons (Kelsey, 2009; Milford, 2020a). This important distinction is often lost on authors who debate personhood. Speaking in the context of ACD, Demarco and Lipuma, for example, touch on this notion when they argue that “a change in personality, even a radical change, does not amount to a new person” (2016, p. 183). Yet they go on to argue that the unique traits of an individual are the basis of their personhood. They state that someone living with dementia:

has the same DNA, many of the same traits, probably similar feelings, and the same brain structure insofar as it is not ravaged by her dementia. She looks the same, has the same social security number and bank accounts, has the same children and grandchildren, and so on. These are all aspects of what it means to be a person. (2016, 183)

It is hard to see why having a specific social security number, or bank account should be associated with someone’s personhood at all – certainly not all persons have social security details or bank accounts. Nevertheless, the point remains: being a person is more than having a particular personality. Recognising this challenge to the Standard View of personhood, there is a growing body of research that affirms a different framework of personhood altogether. This alternate framework, based on a relational understanding of human persons, may offer an approach that is not only philosophically coherent, but pastorally helpful as well.

## Retaining persons through personal relations

Over the last few decades there has been a renewed interest in the question of human personhood from a relational perspective. This has arisen partly as a protest to Western post-enlightenment individualism in philosophies that are more community orientated – for example African Ubuntu Philosophies (Mugumbate & Chereni, 2020) – and has been applied fruitfully to a range of contexts. For example, in discussions around pregnancy loss (Chambers, 2020; Lindemann, 2013; Parsons, 2010) and even authors speaking on dementia itself (Baylis, 2017; Hughes et al., 2006; Kitwood, 1997), this includes in the context of ACDs (Phenwan et al., 2025) . With a foundation in relational ontology (Schaab, 2013), relational views consider personhood as the result of the manner in which certain beings relate to other beings irrespective of their psychological or cognitive characteristics.

In brief, personhood is an “eccentric” concept (Kelsey, 2009). That is to say, what makes a person a person is not something intrinsic to the being itself but “ex-centric” (McFadyen, 1990, p. 27). It is held externally of the respective being in the unique ways other such beings (personal beings) relate to that being: in personal ways. We are persons not because we think, have unique personalities, or are self-reflective, but because we are related to by other persons in personal ways. Persons are created by persons through personal relationships (Milford, 2023; Milford & Shaw, 2023). Consequently, it is the *personal* that comes before the *person* (Kelsey, 2009).

It is best described through the analogy of a new-born infant. Apart from some rudimentary similarities, an infant has none of the characteristics necessary for the Standard View of personhood to apply. It is not rational, self-reflective, nor moral. Yet it is treated as a person within the mother-infant relationship and as such is considered a person (Engelhardt, 1973). In this way, one’s personhood can be held by others (Chambers, 2020; Lindemann, 2013). Consequently, the concept of personhood is relationally ontological because it is predicated on personal relationships and not on substantive qualities. Just like one cannot be a parent without a respective child, nor can one be a daughter without a respective mother (biological or other), so too, one cannot be a person without other persons.

We have explored in-depth the implications of a relational model elsewhere. There we have considered the context of abortion, who can (or cannot) confer personhood and upon which entities, as well as some of the limitations of relational ontology (Milford, 2019, 2020b). Like all models, there are challenges to this construction of personhood. Yet it is arguable that this framework of personhood is not only philosophically coherent but also pastorally helpful.

## The pastoral imperative

Dementia presents numerous serious challenges, but the pastoral challenge should not be underestimated. When one learns of a dementia diagnosis, one inevitably asks: What will happen to me? This is more than simply what will happen physically or psychologically. It is to ask a fundamental question about who we are and what we will become. Here our normative understanding of what we fundamentally are, and what is ultimately of value, will drive our response to dementia. This is true for all those involved: the person living with dementia, their community of loved ones, and the healthcare professionals (HCPs) providing support. It is essential that all involved act from a normative framework that is not only rationally coherent, but pastorally helpful. There is a pastoral imperative – rooted in the existential worry of the possibility of losing oneself or one’s loved one – to seek an understanding of personhood which promotes human flourishing in the face of all that dementia entails (even death).

Some have responded to dementia by ignoring this pastoral imperative. DeGrazia, for example, would have us jettison the notion that “losing our personhood would entail a life not worth living” (DeGrazia, 1999, p. 13). Like him, those that promote the Standard View (including animalism) argue that personhood is simply a phase of life (McMahan, 2002; 2014). To these authors, what is essential is animal biology (Olson, 1997). Yet authors such as these have failed to grasp not only the categorical force of the term *person* but its evaluative force as well (Kelsey, 2009). To be a person is to be included in a category of beings who are evaluated to have universal, inalienable, unqualified dignity and value along with the associated rights. To lose our personhood is to lose this evaluation and to be faced with the prospect of being denigrated to the category of being nothing more than animals (hence the term *animalism)*.

Providing good quality healthcare is more than alleviating physical suffering, especially in the case of dementia where no such cure exists (Kitwood, 1997). Providing healthcare is to promote human flourishing, to seek to restore wholeness (Boudreau & Somerville, 2014). Moving beyond a purely medical understanding of healthcare, writers such as Kitwood (1997) urge us to consider promoting personhood as a way of achieving good care. Here the term personhood is defined as a “standing or status that is bestowed upon one human being, by others, in the context of relationship” (Kitwood, 1997, p. 8). Greenwood et al. (2001), appreciate Kitwood’s move towards meaningful relationships, but they believe Kitwood fails to fully dislodge his understanding of healthcare from a constructivist focus on theories around the subjectivity of the individual patient. Rather, similar to Nwadiugwu (2021), Greenwood et. al. urge dementia care providers to move away from person-centred care models – defined here as individual-patient-centred models – towards a relationship-centred approach suggested by Nolan et al. (2006). Greenwood et. al., taking inspiration from Nolan et al. and Buber’s I-Thou construction, argue against a purely “knowing” view of the patient – whereby the HCP knows about the condition of someone living with dementia – to a focus on preserving the otherness of people living with dementia through relationship and intimacy. This is a challenge all HCPs must face. A small number of HCPs may have been “enculturated in the positivist paradigm” and “attempt to distance themselves and their clinical methods from any priestly role” (Boudreau & Somerville, 2014, p. 26), yet this is an impossibility. The very term “care” is associated with a sense of concern and attention, presupposing a relationship between those giving and receiving care that goes beyond merely a transaction (Greenwood et al., 2001). HCPs cannot simply wash their hands of this pastoral imperative. As many do, HCPs must face the existential threat posed by dementia and provide a response that is not only rationally coherent but pastorally helpful.

It is here argued that, apart from being epistemologically consistent, a relational framework may better address the pastoral imperative than the Standard View in four important ways. (1) A relational view is naturally intuitive: It is intuitive to think that our relationships are fundamental to who we are as people. The phase sortal argument of the Standard View and animalism is counter-intuitive. That someone is now a person but that that they were not always a person at every phase of their life (even though they existed), seems counterintuitive to what many intrinsically believe they are. This is the exact intuition that Olson is attempting to counter in his article *Was I Ever a Fetus* (1997) and what DeGrazia (1999) denies*.* (2) A relational approach is easily comprehensible: It is easy to explain to someone how their personhood is the result of the personal relationships they have with the ones they love and who love them. All involved should be able to follow this simple logic; the person living with dementia, their community of loved ones, and HCPs. This framework can, therefore, easily be shared, even if only at a rudimentary level. However, for those who seek more, the relational framework is also philosophically dense, offering rich deep and meaningful insights into human flourishing (Baylis, 2017; Kelsey, 2009; Lindemann, 2013). The Standard View and animalism, on the other hand, is difficult to comprehend without extensive philosophising. It is hard to easily explain that one’s personal identity is the same as an animal’s personal identity because they both have physical biology. It is hard to understand how one may have a personal identity and yet not be a person. The Standard View requires a sophisticated explanation that HCPs do not often have time for. (3) Relational frameworks are pastorally helpful: To say to someone living with dementia that they will continue to be a person, even if/when confused and irrational, is comforting. To say to a loved one that their mother will always be the same person they always were because they will be loved by the same people is reassuring. Yet to argue that once someone living with dementia has lost key psychological, or cognitive, traits they will cease to be a person is sure to induce further apprehension and fear. Who would want to be told that they will no longer be the person they have always been? (4) A relational model of personhood appeals to many non-western cultures in ways that the standard view does not. As we have noted above, many western societies in the Global North are characterised by an emphasis on the individual (Hofstede, 2001). The debates around individualism vs. collectivism are well known (Maccall, n.d.; Kyriacou, 2016; Soares, 2018). While individualism can help underpin important concepts such as patient autonomy (thereby avoiding paternalism), it often does so at the expense of the community. Many cultures, however, emphasise the importance of the community. For example, within African Ubuntu philosophy, there is a strong sense that what happens to the individual is directly connected with what happens to the community. Epitomised in Mbiti’s adage: “I am because we are”(Mbiti, 1969, p. 106) is the emphasis that the individual cannot be fully comprehended in isolation of the communal personal relationships that are definitive for who they are as persons. A relational – and thereby communal – understanding of personhood, in the context of dementia, can help to ground dementia care in ways that are culturally sensitive to the global care context, and in particular non-western cultures.

## Limitations

There are challenges to a framework of personhood based on relational ontology. An obvious challenge is the legal consequences of a relational framework in the face of end-of life decisions and ACDs. A relational framework offers both opportunities and challenges here which we will have to leave for another time. It is highly probable that a legal definition of personhood is entirely different to a pastoral understanding, and the inter-play between these frameworks would need to be worked out more fully. Consequently, it is possible that a relational view of personhood may guide discussions on ACDs between families and carers while not being applicable to ACDs in legal cases.

Of particular challenge is the philosophy of relational ontology itself – a relatively new concept to many HCPs. On the one hand it is intuitive – relationships are important – yet it is also philosophically dense. Many questions can arise upon first hearing about this framework: What kind of relationships are important? Who can relate to who in person-creating ways? And is it possible to create persons from traditionally non-personal beings (e.g. dogs and cats)? These and other philosophical challenges have been delt with elsewhere. For the HCP it may be challenging to explore all these aspects of relational ontology in a philosophically coherent and rigorous manner – especially in the context of a time-limited medical consultation. Nevertheless, it remains the case that even at a rudimentary level, a relational framework is far more intuitive and easier to understand than the Standard View.

## Conclusions

The pastoral imperative created by dementia should be taken seriously. For many, dementia presents an existential threat to one’s very identity. This is epitomised in Lisa Genova’s seminal novel *Still Alice* (2007) in which the protagonist contracts early onset Alzheimer’s Disease. As the title suggests, a central question for Alice upon her diagnosis is whether she is still Alice when living with dementia. Many have echoed this concern. For example, the feminist author Bayli’s argues emphatically in the affirmative in her analogously titled article *Still Gloria* (2017) *–* she too uses a relational framework. In this article we have argued that the pastoral imperative is best addressed using the relational framework for personhood. This framework is intuitive, rational, and pastorally helpful. For the HCP, its application is similarly intuitive, rational, and pastorally helpful.

Dementia often presents a challenge to human flourishing and our response should address this challenge in practical ways. Consequently, it is important to select a normative framework that has practical applications. When speaking to someone living with dementia, or to any one of their community of loved ones, a HCP should be able to practically respond to the pastoral imperative should it arise. Using a relational framework, should someone express concern about their identity – about who they, or their loved one, will become – the HCP can provide comfort and reassurance by focusing on the importance of personal relationships while living with dementia. In this situation, HCP can focus on the personal relationships that are the basis of personhood. Doing so, the HCP can comfort someone living with dementia by explaining to them that they are not losing themselves or becoming someone else. That someone living with dementia will always be the same person because they are loved by the same people. Likewise, the HCP can offer reassurance to the community of loved ones that they are not now caring for and loving another person. Their loved one will remain the same person through all phases of their life (even this phase marked by dementia) because they remain loved. These words of comfort, framed in relational ways, are practically helpful to HCPs because they are intuitive and easy to understand. Recognising that HCPs often have limited time, the intuitive and simple nature of this normative framework is an immense asset. Comfort can be shared where time is short. On the other hand, when there is time and there is a need for in-depth consultation, the literature is rich and deep for those who wish to really delve into relational ontology. Consequently, a relational framework offers the HCP the flexibility to share short words of comfort, or to take their time to explore the significance of deep personal relationships for those living with dementia.

## Data Availability

Data sharing is not applicable to this article as no datasets were generated or analysed during the current study.
